# Bis(1,10-phenanthroline-κ^2^ *N*,*N*′)(sulfato-κ^2^ *O*,*O*′)nickel(II) ethane-1,2-diol solvate

**DOI:** 10.1107/S1600536809026269

**Published:** 2009-07-11

**Authors:** Kai-Long Zhong, Chao Ni, Jian-Mei Wang

**Affiliations:** a Department of Applied Chemistry, Nanjing College of Chemical Technology, Nanjing, Jiangsu Province, People’s Republic of China

## Abstract

In the title compound, [Ni(SO_4_)(C_12_H_8_N_2_)_2_]·C_2_H_6_O_2_, the coordination polyhedron around the Ni^2+^ ion is a distorted octahedron, with four N atoms from two phenanthroline groups and two O atoms from a bidentate sulfate ligand. The Ni^2+^ ion lies on a special position of site symmetry 2. Inter­molecular O—H⋯O hydrogen bonds help to stabilize the structure. The OH group of the ethane-1,2-diol solvent is disordered over two positions with equal occupancy.

## Related literature

For Ni–phen complexes with chloride anions and water mol­ecules as a second ligand, see: Chen *et al.* (2005 ▶); Su & Xu (2005 ▶); Tang *et al.* (2007 ▶). For isostructural compounds, see: Zhong *et al.* (2006 ▶); Lu *et al.* (2006 ▶); Zhu *et al.* (2006*a* ▶,*b* ▶).
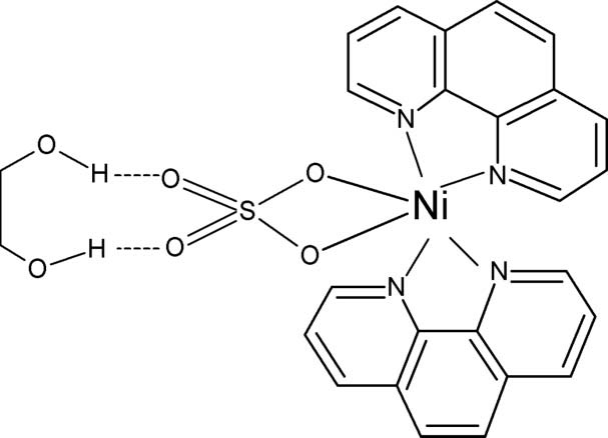


## Experimental

### 

#### Crystal data

[Ni(SO_4_)(C_12_H_8_N_2_)_2_]·C_2_H_6_O_2_
*M* *_r_* = 577.25
Monoclinic, 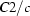

*a* = 18.4551 (9) Å
*b* = 11.8839 (5) Å
*c* = 12.7526 (6) Å
β = 118.991 (6)°
*V* = 2446.4 (2) Å^3^
*Z* = 4
Mo *K*α radiation
μ = 0.93 mm^−1^
*T* = 295 K
0.36 × 0.33 × 0.28 mm

#### Data collection

Oxford Diffraction Gemini S Ultra diffractometer
Absorption correction: multi-scan (*ABSPACK*; Oxford Diffraction, 2009 ▶) *T*_min_ = 0.731, *T*_max_ = 0.781
11586 measured reflections
3010 independent reflections
2467 reflections with *I* > 2σ(*I*)
*R*_int_ = 0.029

#### Refinement

*R*[*F* ^2^ > 2σ(*F* ^2^)] = 0.036
*wR*(*F* ^2^) = 0.106
*S* = 1.08
3010 reflections
183 parameters
17 restraints
H-atom parameters constrained
Δρ_max_ = 0.35 e Å^−3^
Δρ_min_ = −0.52 e Å^−3^

### 

Data collection: *CrysAlisPro* (Oxford Diffraction, 2009 ▶); cell refinement: *CrysAlisPro*; data reduction: *CrysAlisPro*; program(s) used to solve structure: *SHELXS97* (Sheldrick, 2008 ▶); program(s) used to refine structure: *SHELXL97* (Sheldrick, 2008 ▶); molecular graphics: *XP* in *SHELXTL* (Sheldrick, 2008 ▶); software used to prepare material for publication: *SHELXTL*.

## Supplementary Material

Crystal structure: contains datablocks global, I. DOI: 10.1107/S1600536809026269/pk2166sup1.cif

Structure factors: contains datablocks I. DOI: 10.1107/S1600536809026269/pk2166Isup2.hkl

Additional supplementary materials:  crystallographic information; 3D view; checkCIF report

## Figures and Tables

**Table 1:** Hydrogen-bond geometry (Å, °)

*D*—H⋯*A*	*D*—H	H⋯*A*	*D*⋯*A*	*D*—H⋯*A*
O3—H3*A*⋯O1	0.82	2.15	2.659 (7)	121
O3′—H3′⋯O1	0.82	2.47	2.763 (5)	102

**Fig. 1.**
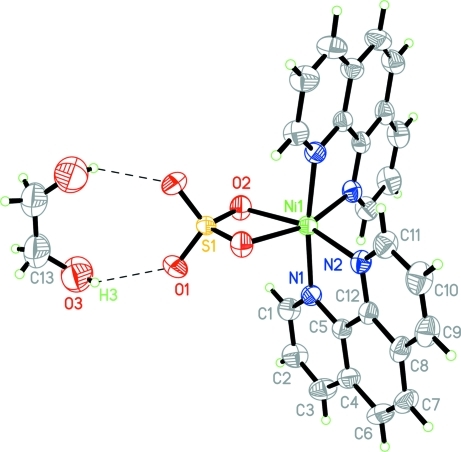
The molecular structure showing the atom-numbering scheme and with displacement ellipsoids drawn at the 50% probability level. The dashed lines represent O—H···O interactions. Unlabeled atoms are related to the labelled atoms by the symmetry operator (-x, y, -z +3/2). Only one disorder component is shown.

## supplementary crystallographic information

### Comment

Ni-phen (phen = phenanthroline) complexes with chloride-anion and water-molecule ligands have been synthesized and characterized by X-ray diffraction (Chen *et al.*, 2005; Su & Xu, 2005; Tang *et al.*, 2007).  The title nickel complex [NiSO_4_(phen)_2_].C_2_H_6_O_2_, Fig. 1, is isostructural to the recently reported cobalt(II) and cadmium(II) analogs (Zhong *et al.*, 2006; Lu *et al.*, 2006). A twofold rotation axis passes through the Ni and S atoms, and also through the mid-point of the C—C bond of the solvent molcule. The Ni^II^ center has an octahedral geometry, with four N atoms from two phen groups and two O atoms from a bidentate sulfate ligand. The geometry of the phen and sulfate ligands are in good agreement with those reported in the two isomorphous complexes [ZnSO_4_(phen)_2_].C_2_H_6_O_2_ and [MnSO_4_(phen)_2_].C_2_H_6_O_2_ (Zhu *et al.*, 2006a,b).

The ethane-1,2-diol solvent is disordered over two positions, and is hydrogen bonded to the sulfate ligand (Table 1).

### Experimental

Green block-shaped crystals of the title compound were obtained by a procedure similar to that described previously (Zhong *et al.*, 2006), but with NiSO_4_.7H_2_O in place of CoSO_4_.7H_2_O.

### Refinement

The H atoms of phen were positioned geometrically and allowed to ride on their parent atoms, with C—H = 0.93 Å and *U*_iso_(H) = 1.2*U*_eq_(C). The O atom of the ethane-1,2-diol solvent is disordered over two positions with site-occupancy factors of 1/2, sharing a common atom C13. The C13—C13 ^i^ (i = -*x*, *y*, -*z*+3/2), C13—O3 and C13—O3' distances were restrained to 1.501 (4), 1.304 (5) and 1.339 (5) Å, respectively. The H atoms of the ethane-1,2-diol were located in a difference map and then allowed to ride on their parent atoms, with C—H = 0.97 Å and O—H = 0.82 Å; *U*_iso_(H) = 1.2*U*_eq_(C) and 1.5*U*_eq_(O).

### Crystal data

**Table:**

[Ni(SO_4_)(C_12_H_8_N_2_)_2_]·C_2_H_6_O_2_	*F*(000) = 1192
*M**_r_* = 577.25	*D*_x_ = 1.567 Mg m^−^^3^
Monoclinic, *C*2/*c*	Mo *K*α radiation, λ = 0.71073 Å
Hall symbol: -C 2yc	Cell parameters from 6902 reflections
*a* = 18.4551 (9) Å	θ = 3.2–30.6°
*b* = 11.8839 (5) Å	µ = 0.93 mm^−^^1^
*c* = 12.7526 (6) Å	*T* = 295 K
β = 118.991 (6)°	Block, green
*V* = 2446.4 (2) Å^3^	0.36 × 0.33 × 0.28 mm
*Z* = 4	

### Data collection

**Table:**

Oxford Diffraction Gemini S Ultra diffractometer	3010 independent reflections
Radiation source: fine-focus sealed tube	2467 reflections with *I* > 2σ(*I*)
graphite	*R*_int_ = 0.029
Detector resolution: 8.1241 pixels mm^-1^	θ_max_ = 28.3°, θ_min_ = 3.2°
φ and ω scans	*h* = −24→24
Absorption correction: multi-scan (*ABSPACK*; Oxford Diffraction, 2009)	*k* = −15→15
*T*_min_ = 0.731, *T*_max_ = 0.781	*l* = −17→16
11586 measured reflections	

### Refinement

**Table:**

Refinement on *F*^2^	Secondary atom site location: difference Fourier map
Least-squares matrix: full	Hydrogen site location: inferred from neighbouring sites
*R*[*F*^2^ > 2σ(*F*^2^)] = 0.036	H-atom parameters constrained
*wR*(*F*^2^) = 0.106	*w* = 1/[σ^2^(*F*_o_^2^) + (0.0643*P*)^2^ + 0.296*P*] where *P* = (*F*_o_^2^ + 2*F*_c_^2^)/3
*S* = 1.08	(Δ/σ)_max_ < 0.001
3010 reflections	Δρ_max_ = 0.35 e Å^−^^3^
183 parameters	Δρ_min_ = −0.52 e Å^−^^3^
17 restraints	Extinction correction: *SHELXL97* (Sheldrick, 2008), Fc^*^=kFc[1+0.001xFc^2^λ^3^/sin(2θ)]^-1/4^
Primary atom site location: structure-invariant direct methods	Extinction coefficient: 0.0055 (6)

### Special details

**Table:**

Geometry. All e.s.d.'s (except the e.s.d. in the dihedral angle between two l.s. planes) are estimated using the full covariance matrix. The cell e.s.d.'s are taken into account individually in the estimation of e.s.d.'s in distances, angles and torsion angles; correlations between e.s.d.'s in cell parameters are only used when they are defined by crystal symmetry. An approximate (isotropic) treatment of cell e.s.d.'s is used for estimating e.s.d.'s involving l.s. planes.
Refinement. Refinement of *F*^2^ against ALL reflections. The weighted *R*-factor *wR* and goodness of fit *S* are based on *F*^2^, conventional *R*-factors *R* are based on *F*, with *F* set to zero for negative *F*^2^. The threshold expression of *F*^2^ > σ(*F*^2^) is used only for calculating *R*-factors(gt) *etc*. and is not relevant to the choice of reflections for refinement. *R*-factors based on *F*^2^ are statistically about twice as large as those based on *F*, and *R*- factors based on ALL data will be even larger.

### Fractional atomic coordinates and isotropic or equivalent isotropic displacement parameters (Å^2^)

**Table:**

	*x*	*y*	*z*	*U*_iso_*/*U*_eq_	Occ. (<1)
Ni1	0.0000	0.19859 (3)	0.7500	0.02868 (15)	
S1	0.0000	−0.02657 (6)	0.7500	0.02857 (19)	
O2	−0.05633 (9)	0.05119 (13)	0.65249 (13)	0.0379 (4)	
O1	0.04687 (11)	−0.09628 (15)	0.71006 (17)	0.0479 (5)	
N1	0.07956 (11)	0.21105 (15)	0.67829 (16)	0.0314 (4)	
N2	0.08892 (12)	0.30656 (14)	0.87419 (16)	0.0331 (4)	
C5	0.14702 (13)	0.27545 (18)	0.74544 (17)	0.0291 (4)	
C7	0.28779 (15)	0.4016 (2)	0.8974 (2)	0.0438 (6)	
H7	0.3347	0.4428	0.9481	0.053*	
C9	0.22836 (17)	0.4292 (2)	1.0353 (2)	0.0458 (6)	
H9	0.2743	0.4702	1.0893	0.055*	
C4	0.21016 (14)	0.2934 (2)	0.7163 (2)	0.0368 (5)	
C8	0.22400 (15)	0.38596 (19)	0.92872 (19)	0.0368 (5)	
C12	0.15344 (13)	0.32477 (17)	0.85260 (18)	0.0303 (5)	
C6	0.28132 (15)	0.3577 (2)	0.7956 (2)	0.0454 (6)	
H6	0.3236	0.3694	0.7769	0.055*	
C1	0.07228 (15)	0.1648 (2)	0.5783 (2)	0.0391 (5)	
H1	0.0260	0.1209	0.5310	0.047*	
C2	0.13218 (17)	0.1802 (2)	0.5424 (2)	0.0474 (6)	
H2	0.1250	0.1481	0.4715	0.057*	
C3	0.20060 (16)	0.2421 (2)	0.6110 (2)	0.0475 (6)	
H3	0.2413	0.2506	0.5885	0.057*	
C10	0.16452 (19)	0.4099 (2)	1.0571 (2)	0.0507 (7)	
H10	0.1667	0.4375	1.1268	0.061*	
C11	0.09572 (16)	0.3488 (2)	0.9757 (2)	0.0428 (6)	
H11	0.0527	0.3369	0.9927	0.051*	
C13	0.0291 (3)	−0.4047 (3)	0.7245 (4)	0.0978 (14)	
H13A	0.0809	−0.4351	0.7869	0.117*	
H13B	0.0073	−0.4600	0.6606	0.117*	
O3	0.0492 (5)	−0.3177 (5)	0.6817 (6)	0.098 (2)	0.50
H3A	0.0752	−0.2727	0.7359	0.147*	0.50
O3'	0.0850 (3)	−0.3224 (4)	0.7512 (6)	0.0726 (17)	0.50
H3'	0.0792	−0.2939	0.6891	0.109*	0.50

### Atomic displacement parameters (Å^2^)

**Table:**

	*U*^11^	*U*^22^	*U*^33^	*U*^12^	*U*^13^	*U*^23^
Ni1	0.0250 (2)	0.0289 (2)	0.0293 (2)	0.000	0.01088 (16)	0.000
S1	0.0222 (3)	0.0282 (4)	0.0318 (4)	0.000	0.0103 (3)	0.000
O2	0.0302 (8)	0.0346 (9)	0.0318 (8)	−0.0013 (7)	0.0014 (6)	−0.0005 (6)
O1	0.0430 (10)	0.0455 (11)	0.0622 (11)	0.0066 (8)	0.0310 (9)	−0.0055 (8)
N1	0.0282 (9)	0.0318 (10)	0.0304 (9)	−0.0019 (8)	0.0111 (7)	−0.0032 (7)
N2	0.0377 (10)	0.0293 (10)	0.0325 (9)	−0.0029 (8)	0.0172 (8)	−0.0026 (7)
C5	0.0266 (10)	0.0280 (11)	0.0282 (9)	0.0013 (8)	0.0097 (8)	0.0015 (8)
C7	0.0300 (12)	0.0428 (15)	0.0448 (13)	−0.0097 (10)	0.0072 (10)	−0.0017 (10)
C9	0.0495 (15)	0.0406 (14)	0.0351 (12)	−0.0087 (12)	0.0109 (11)	−0.0088 (10)
C4	0.0307 (11)	0.0397 (13)	0.0387 (12)	−0.0003 (10)	0.0157 (9)	0.0034 (9)
C8	0.0375 (12)	0.0297 (12)	0.0326 (11)	−0.0042 (10)	0.0088 (9)	−0.0003 (9)
C12	0.0300 (11)	0.0267 (11)	0.0278 (10)	−0.0001 (8)	0.0091 (8)	0.0013 (8)
C6	0.0295 (11)	0.0544 (17)	0.0491 (14)	−0.0070 (12)	0.0164 (10)	0.0007 (12)
C1	0.0381 (12)	0.0429 (13)	0.0336 (11)	−0.0037 (11)	0.0154 (10)	−0.0085 (9)
C2	0.0504 (15)	0.0572 (17)	0.0388 (13)	−0.0021 (13)	0.0248 (12)	−0.0087 (11)
C3	0.0428 (14)	0.0621 (18)	0.0453 (13)	−0.0046 (13)	0.0275 (12)	−0.0026 (12)
C10	0.0664 (18)	0.0476 (16)	0.0360 (12)	−0.0080 (13)	0.0231 (12)	−0.0121 (11)
C11	0.0522 (15)	0.0417 (14)	0.0386 (12)	−0.0074 (12)	0.0252 (11)	−0.0086 (10)
C13	0.111 (3)	0.066 (2)	0.141 (3)	0.002 (2)	0.081 (3)	−0.002 (2)
O3	0.130 (5)	0.087 (4)	0.115 (4)	0.009 (3)	0.090 (4)	0.017 (3)
O3'	0.061 (3)	0.042 (3)	0.129 (5)	0.017 (2)	0.056 (3)	0.028 (3)

### Geometric parameters (Å, °)

**Table:**

Ni1—N1^i^	2.0774 (18)	C9—C8	1.418 (3)
Ni1—N1	2.0774 (18)	C9—H9	0.9300
Ni1—N2	2.0805 (19)	C4—C3	1.406 (3)
Ni1—N2^i^	2.0805 (18)	C4—C6	1.430 (3)
Ni1—O2	2.1077 (16)	C8—C12	1.393 (3)
Ni1—O2^i^	2.1077 (16)	C6—H6	0.9300
Ni1—S1	2.6757 (8)	C1—C2	1.398 (3)
S1—O1^i^	1.4563 (17)	C1—H1	0.9300
S1—O1	1.4563 (17)	C2—C3	1.352 (4)
S1—O2^i^	1.4926 (16)	C2—H2	0.9300
S1—O2	1.4926 (16)	C3—H3	0.9300
N1—C1	1.334 (3)	C10—C11	1.392 (4)
N1—C5	1.355 (3)	C10—H10	0.9300
N2—C11	1.337 (3)	C11—H11	0.9300
N2—C12	1.363 (3)	C13—O3	1.304 (5)
C5—C4	1.401 (3)	C13—O3'	1.339 (5)
C5—C12	1.438 (3)	C13—C13^i^	1.501 (4)
C7—C6	1.349 (4)	C13—H13A	0.9700
C7—C8	1.427 (4)	C13—H13B	0.9700
C7—H7	0.9300	O3—H3A	0.8200
C9—C10	1.355 (4)	O3'—H3'	0.8200
			
N1^i^—Ni1—N1	171.82 (10)	C8—C7—H7	119.4
N1^i^—Ni1—N2	94.93 (7)	C10—C9—C8	119.0 (2)
N1—Ni1—N2	79.99 (7)	C10—C9—H9	120.5
N1^i^—Ni1—N2^i^	79.99 (7)	C8—C9—H9	120.5
N1—Ni1—N2^i^	94.93 (7)	C5—C4—C3	116.9 (2)
N2—Ni1—N2^i^	103.84 (10)	C5—C4—C6	119.4 (2)
N1^i^—Ni1—O2	93.85 (7)	C3—C4—C6	123.7 (2)
N1—Ni1—O2	92.94 (7)	C12—C8—C9	117.2 (2)
N2—Ni1—O2	160.60 (7)	C12—C8—C7	119.5 (2)
N2^i^—Ni1—O2	94.70 (6)	C9—C8—C7	123.3 (2)
N1^i^—Ni1—O2^i^	92.94 (7)	N2—C12—C8	123.7 (2)
N1—Ni1—O2^i^	93.85 (7)	N2—C12—C5	116.71 (19)
N2—Ni1—O2^i^	94.70 (6)	C8—C12—C5	119.6 (2)
N2^i^—Ni1—O2^i^	160.60 (7)	C7—C6—C4	120.7 (2)
O2—Ni1—O2^i^	67.58 (8)	C7—C6—H6	119.7
N1^i^—Ni1—S1	94.09 (5)	C4—C6—H6	119.7
N1—Ni1—S1	94.09 (5)	N1—C1—C2	122.0 (2)
N2—Ni1—S1	128.08 (5)	N1—C1—H1	119.0
N2^i^—Ni1—S1	128.08 (5)	C2—C1—H1	119.0
O2—Ni1—S1	33.79 (4)	C3—C2—C1	119.9 (2)
O2^i^—Ni1—S1	33.79 (4)	C3—C2—H2	120.1
O1^i^—S1—O1	110.66 (15)	C1—C2—H2	120.1
O1^i^—S1—O2^i^	110.65 (10)	C2—C3—C4	119.9 (2)
O1—S1—O2^i^	110.59 (10)	C2—C3—H3	120.0
O1^i^—S1—O2	110.59 (10)	C4—C3—H3	120.0
O1—S1—O2	110.65 (10)	C9—C10—C11	120.3 (2)
O2^i^—S1—O2	103.50 (13)	C9—C10—H10	119.9
O1^i^—S1—Ni1	124.67 (8)	C11—C10—H10	119.9
O1—S1—Ni1	124.67 (8)	N2—C11—C10	122.8 (2)
O2^i^—S1—Ni1	51.75 (6)	N2—C11—H11	118.6
O2—S1—Ni1	51.75 (6)	C10—C11—H11	118.6
S1—O2—Ni1	94.46 (7)	O3—C13—O3'	35.7 (4)
C1—N1—C5	118.22 (19)	O3—C13—C13^i^	126.2 (4)
C1—N1—Ni1	128.76 (16)	O3'—C13—C13^i^	121.0 (4)
C5—N1—Ni1	113.02 (13)	O3—C13—H13A	105.8
C11—N2—C12	117.1 (2)	O3'—C13—H13A	74.5
C11—N2—Ni1	129.66 (16)	C13^i^—C13—H13A	105.8
C12—N2—Ni1	112.83 (14)	O3—C13—H13B	105.8
N1—C5—C4	123.10 (19)	O3'—C13—H13B	131.5
N1—C5—C12	117.27 (18)	C13^i^—C13—H13B	105.8
C4—C5—C12	119.6 (2)	H13A—C13—H13B	106.2
C6—C7—C8	121.2 (2)	C13—O3—H3A	109.5
C6—C7—H7	119.4	C13—O3'—H3'	109.5
			
N1^i^—Ni1—S1—O1^i^	0.87 (10)	O2^i^—Ni1—N2—C11	−82.5 (2)
N1—Ni1—S1—O1^i^	−179.13 (10)	S1—Ni1—N2—C11	−88.3 (2)
N2—Ni1—S1—O1^i^	100.39 (11)	N1^i^—Ni1—N2—C12	−177.26 (15)
N2^i^—Ni1—S1—O1^i^	−79.61 (11)	N1—Ni1—N2—C12	−3.72 (14)
O2—Ni1—S1—O1^i^	−89.95 (12)	N2^i^—Ni1—N2—C12	−96.38 (15)
O2^i^—Ni1—S1—O1^i^	90.05 (12)	O2—Ni1—N2—C12	66.1 (3)
N1^i^—Ni1—S1—O1	−179.13 (10)	O2^i^—Ni1—N2—C12	89.37 (15)
N1—Ni1—S1—O1	0.87 (10)	S1—Ni1—N2—C12	83.62 (15)
N2—Ni1—S1—O1	−79.61 (11)	C1—N1—C5—C4	−1.6 (3)
N2^i^—Ni1—S1—O1	100.39 (11)	Ni1—N1—C5—C4	178.30 (17)
O2—Ni1—S1—O1	90.05 (12)	C1—N1—C5—C12	179.8 (2)
O2^i^—Ni1—S1—O1	−89.95 (12)	Ni1—N1—C5—C12	−0.3 (2)
N1^i^—Ni1—S1—O2^i^	−89.18 (9)	N1—C5—C4—C3	0.8 (3)
N1—Ni1—S1—O2^i^	90.82 (9)	C12—C5—C4—C3	179.4 (2)
N2—Ni1—S1—O2^i^	10.34 (10)	N1—C5—C4—C6	−176.7 (2)
N2^i^—Ni1—S1—O2^i^	−169.66 (10)	C12—C5—C4—C6	1.9 (3)
O2—Ni1—S1—O2^i^	180.0	C10—C9—C8—C12	−0.4 (4)
N1^i^—Ni1—S1—O2	90.82 (9)	C10—C9—C8—C7	−180.0 (2)
N1—Ni1—S1—O2	−89.18 (9)	C6—C7—C8—C12	−0.3 (4)
N2—Ni1—S1—O2	−169.66 (10)	C6—C7—C8—C9	179.2 (3)
N2^i^—Ni1—S1—O2	10.34 (10)	C11—N2—C12—C8	−1.3 (3)
O2^i^—Ni1—S1—O2	180.0	Ni1—N2—C12—C8	−174.28 (17)
O1^i^—S1—O2—Ni1	118.53 (10)	C11—N2—C12—C5	177.7 (2)
O1—S1—O2—Ni1	−118.49 (9)	Ni1—N2—C12—C5	4.7 (2)
O2^i^—S1—O2—Ni1	0.0	C9—C8—C12—N2	1.2 (3)
N1^i^—Ni1—O2—S1	−91.59 (8)	C7—C8—C12—N2	−179.2 (2)
N1—Ni1—O2—S1	92.96 (8)	C9—C8—C12—C5	−177.8 (2)
N2—Ni1—O2—S1	25.2 (2)	C7—C8—C12—C5	1.8 (3)
N2^i^—Ni1—O2—S1	−171.85 (8)	N1—C5—C12—N2	−3.0 (3)
O2^i^—Ni1—O2—S1	0.0	C4—C5—C12—N2	178.32 (19)
N1^i^—Ni1—N1—C1	−126.0 (2)	N1—C5—C12—C8	176.05 (19)
N2—Ni1—N1—C1	−178.0 (2)	C4—C5—C12—C8	−2.6 (3)
N2^i^—Ni1—N1—C1	−74.8 (2)	C8—C7—C6—C4	−0.4 (4)
O2—Ni1—N1—C1	20.2 (2)	C5—C4—C6—C7	−0.4 (4)
O2^i^—Ni1—N1—C1	87.9 (2)	C3—C4—C6—C7	−177.7 (3)
S1—Ni1—N1—C1	54.0 (2)	C5—N1—C1—C2	0.6 (4)
N1^i^—Ni1—N1—C5	54.20 (14)	Ni1—N1—C1—C2	−179.27 (19)
N2—Ni1—N1—C5	2.17 (14)	N1—C1—C2—C3	1.2 (4)
N2^i^—Ni1—N1—C5	105.39 (15)	C1—C2—C3—C4	−1.9 (4)
O2—Ni1—N1—C5	−159.64 (15)	C5—C4—C3—C2	1.0 (4)
O2^i^—Ni1—N1—C5	−91.93 (15)	C6—C4—C3—C2	178.3 (3)
S1—Ni1—N1—C5	−125.80 (14)	C8—C9—C10—C11	−0.3 (4)
N1^i^—Ni1—N2—C11	10.8 (2)	C12—N2—C11—C10	0.5 (4)
N1—Ni1—N2—C11	−175.6 (2)	Ni1—N2—C11—C10	172.17 (19)
N2^i^—Ni1—N2—C11	91.7 (2)	C9—C10—C11—N2	0.2 (4)
O2—Ni1—N2—C11	−105.8 (3)		

Symmetry codes: (i) −*x*, *y*, −*z*+3/2.

### Hydrogen-bond geometry (Å, °)

**Table:**

*D*—H···*A*	*D*—H	H···*A*	*D*···*A*	*D*—H···*A*
O3—H3A···O1	0.82	2.15	2.659 (7)	121
O3'—H3'···O1	0.82	2.47	2.763 (5)	102
